# Stimulating Preconception Care Uptake by Women With a Vulnerable Health Status Through a Mobile Health App (Pregnant Faster): Pilot Feasibility Study

**DOI:** 10.2196/53614

**Published:** 2024-04-22

**Authors:** Sharissa M Smith, Babette Bais, Hafez Ismaili M'hamdi, Maartje HN Schermer, Régine PM Steegers-Theunissen

**Affiliations:** 1 Department of Obstetrics and Gynecology Erasmus University Medical Center Rotterdam Netherlands; 2 Department of Medical Ethics, Philosophy and History of Medicine Erasmus University Medical Center Rotterdam Netherlands

**Keywords:** preconception care, mHealth, mobile health, pregnancy preparation, nudge, health inequality, socioeconomic status, lifestyle, women, pregnancy, pregnant women, pregnant, socioeconomic, pilot feasibility study, mHealth app, mHealth application, app, application, risk factor, nutrition, stress, chronic stress, health literacy, usability, user satisfaction, user, users

## Abstract

**Background:**

A low socioeconomic status is associated with a vulnerable health status (VHS) through the accumulation of health-related risk factors, such as poor lifestyle behaviors (eg, inadequate nutrition, chronic stress, and impaired health literacy). For pregnant women, a VHS translates into a high incidence of adverse pregnancy outcomes and therefore pregnancy-related inequity. We hypothesize that stimulating adequate pregnancy preparation, targeting lifestyle behaviors and preconception care (PCC) uptake, can reduce these inequities and improve the pregnancy outcomes of women with a VHS. A nudge is a behavioral intervention aimed at making healthy choices easier and more attractive and may therefore be a feasible way to stimulate engagement in pregnancy preparation and PCC uptake, especially in women with a VHS. To support adequate pregnancy preparation, we designed a mobile health (mHealth) app, Pregnant Faster, that fits the preferences of women with a VHS and uses nudging to encourage PCC consultation visits and engagement in education on healthy lifestyle behaviors.

**Objective:**

This study aimed to test the feasibility of Pregnant Faster by determining usability and user satisfaction, the number of visited PCC consultations, and the course of practical study conduction.

**Methods:**

Women aged 18-45 years, with low-to-intermediate educational attainment, who were trying to become pregnant within 12 months were included in this open cohort. Recruitment took place through social media, health care professionals, and distribution of flyers and posters from September 2021 until June 2022. Participants used Pregnant Faster daily for 4 weeks, earning coins by reading blogs on pregnancy preparation, filling out a daily questionnaire on healthy lifestyle choices, and registering for a PCC consultation with a midwife. Earned coins could be spent on rewards, such as fruit, mascara, and baby products. Evaluation took place through the mHealth App Usability Questionnaire (MAUQ), an additional interview or questionnaire, and assessment of overall study conduction.

**Results:**

Due to limited inclusions, the inclusion criterion “living in a deprived neighborhood” was dropped. This resulted in the inclusion of 47 women, of whom 39 (83%) completed the intervention. In total, 16 (41%) of 39 participants visited a PCC consultation, with their main motivation being obtaining personalized information. The majority of participants agreed with 16 (88.9%) of 18 statements of the MAUQ, indicating high user satisfaction. The mean rating was 7.7 (SD 1.0) out of 10. Points of improvement included recruitment of the target group, simplification of the log-in system, and automation of manual tasks.

**Conclusions:**

Nudging women through Pregnant Faster to stimulate pregnancy preparation and PCC uptake has proven feasible, but the inclusion criteria must be revised. A substantial number of PCC consultations were conducted, and this study will therefore be continued with an open cohort of 400 women, aiming to establish the (cost-)effectiveness of an updated version, named Pregnant Faster 2.

**International Registered Report Identifier (IRRID):**

RR2-10.2196/45293

## Introduction

A low socioeconomic status (SES) is associated with a vulnerable health status (VHS), which research suggests is grounded in the accumulation of risk factors, such as inadequate nutrition, smoking, and increased mental stressors [1-4]. For women, a low SES means they are more likely to have a VHS, which translates into a higher incidence of adverse pregnancy outcomes in this group [5-9]. These adverse outcomes originate at least partly in the periconception period [10], during which gametogenesis, embryonic development, and placentation take place, laying the foundation for perinatal outcomes, as well as the child’s lifelong health [11,12]. For example, an accumulation of 2 or more maternal risk factors impacts embryonic growth [13], which is associated with midpregnancy fetal weight and birth weight [14]. In addition, infants born small for their gestational age are more susceptible to noncommunicable diseases, such as diabetes mellitus and cardiovascular disease [15]. The effects of adverse pregnancy outcomes therefore hit twice: once in utero and once in later life. This increases the child’s chance of a VHS in adulthood, which, once again, may influence pregnancy outcomes. These transgenerational effects are further maintained by impaired health agency, which is associated with a low SES and diminishes the likelihood of seeking necessary care [16]. In accordance with these findings, research shows that women with a VHS are less likely to engage in pregnancy preparation and take up preconception care (PCC) [17].

PCC is usually given by a midwife or an obstetrician and is aimed at identification of possible risk factors for adverse outcomes, ameliorating those that are modifiable prior to pregnancy [18]. This includes adopting healthy lifestyle behaviors and making beneficial choices in general that will increase the chance of having a healthy pregnancy and baby. Although ≥80% of women who wish to become pregnant have at least 1 modifiable risk factor for adverse pregnancy outcomes [19,20], the uptake of PCC remains low due to insufficient awareness of risk factors and the benefits of PCC [21]. Women with a low SES may encounter additional barriers when engaging in pregnancy preparation, as they are already burdened by the deprived circumstances in which they live. Supporting this group by making pregnancy preparation easier and attractive might be a suitable way to relieve the inequity regarding their pregnancy outcomes.

Our research group has previously developed the web-based PCC tool Smarter Pregnancy, an interactive, tailored, mobile health (mHealth) platform that offers practical coaching and customized feedback on nutrition and other lifestyle behaviors of prospective parents [22]. Smarter Pregnancy has proven to be effective in supporting healthy choices in women with a VHS, in addition to being valued highly by them [23]. To further support PCC engagement in women with a VHS, we have designed an mHealth app that especially fits their needs and preferences [24]: the app-based nudge Pregnant Faster. A nudge is an intervention that stimulates making beneficial choices by increasing the attractiveness and easiness of healthy behavior [25]. An in-depth explanation of nudge theory and its application in health policy can be found in the study by Murayama et al [26].

In the case of Pregnant Faster, participants are nudged through a loyalty program that entails collecting coins by engaging with the app and ordering rewards using those coins. The design of Pregnant Faster can be viewed as a macrolevel nudge, containing multiple microlevel nudges aimed at stimulating pregnancy preparation and encouraging the uptake of PCC. For example, the monetary value of a coin is a microlevel nudge; it varies from €0.06 to €0.26 (US $0.06-$0.26), depending on the type of reward. Healthy rewards, such as folic acid supplements, are relatively cheap, steering participants toward picking them over luxury goods, while maintaining their freedom to choose. The most important feature of the app, which also yields the highest number of coins, is the possibility to register for a PCC consultation with a nearby midwife, promoting blended care: an effective way to promote pregnancy preparation [27]. As midwives are the primary health care providers for pregnant women in the Netherlands, PCC consultations are beneficial for the bond between health care provider and client prior to and during pregnancy. The full description of Pregnant Faster’s design process, detailing the imbedded nudges, has been published in *JMIR Protocols* [28].

**Figure 1:**
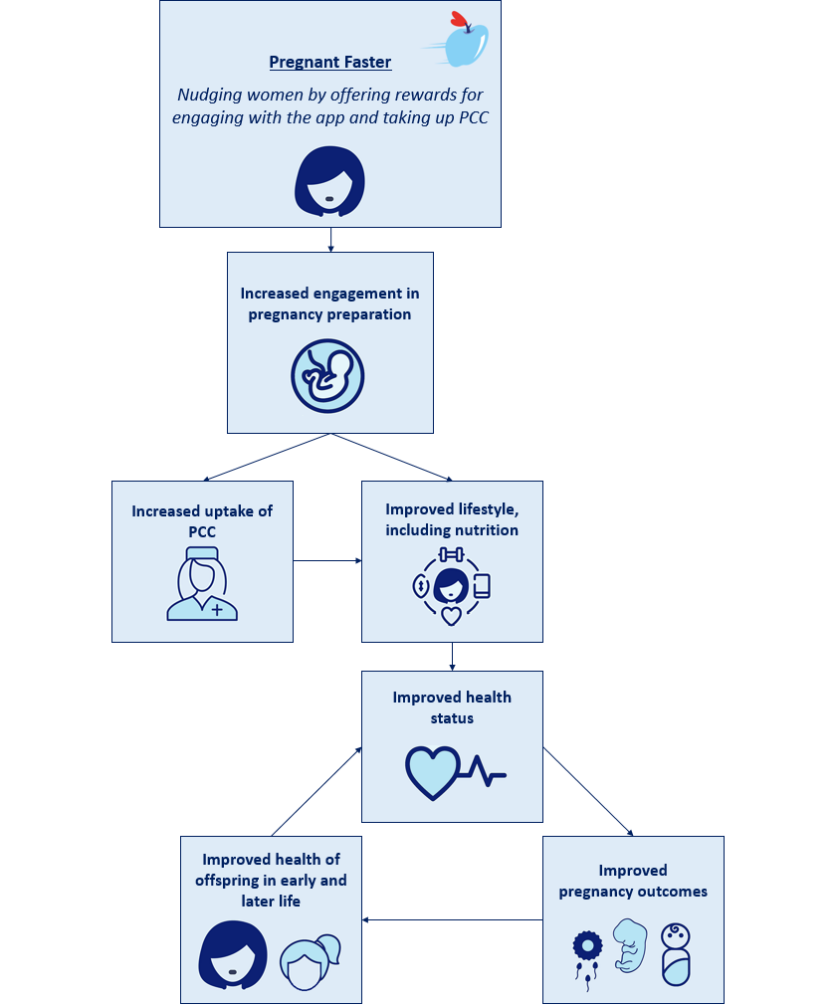
Aim of the mHealth app Pregnant Faster. mHealth: mobile health; PCC: preconception care.

The aim of this pilot study was to determine Pregnant Faster’s feasibility pertaining to usability and user satisfaction, the number of PCC consultations booked and visited by participants, and the course of practical conduction regarding the inclusion process, reward allocation, and finalization of the study. In addition, the results of this study will be used to further develop Pregnant Faster and lay the foundation for a larger cohort study to establish its (cost-)effectiveness. Our overall ambition is that Pregnant Faster contributes to the improvement of short-term and long-term health in mothers with a VHS, their children, and future generations (Figure 1).

## Methods

### Ethical Considerations

This study was assessed and approved by the Medical Ethical Committee of the Erasmus University Medical Center, Rotterdam, The Netherlands (MEC-2020-0974). Informed consent was obtained from all participants via email. Considering the low risk of this study, composing a Data Safety Monitoring Board was deemed unnecessary.

### Recruitment and Inclusion

**Figure 2:**
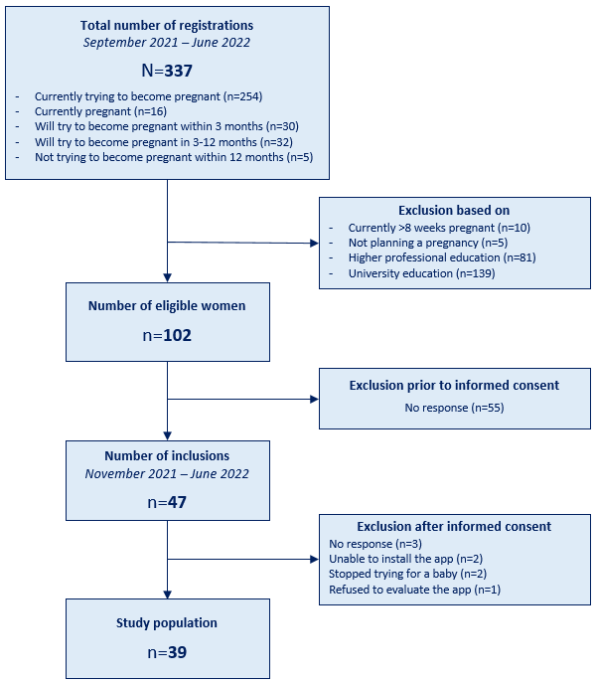
Inclusion flowchart.

Our aim was to include 40 participants in this study. Between September 2021 and June 2022, 337 women registered for this study, of which 102 (30.3%) were eligible for inclusion. Due to a higher-than-expected confidentiality, integrity, and availability (CIA) Triad classification (a risk score regarding user information safety [29]), additional security demands were necessary. Fulfilling these demands delayed the launch of Pregnant Faster from September 2021 to November 2021, leading to a loss of recruited eligible women. From the launch onward, 47 (46.1%) women were included, of which 39 (83%) completed the intervention (Figure 2).

Recruitment took place through the Sneller Zwanger website [30], which was distributed through posters and flyers, advertisements on the social media platforms Facebook and Instagram, and midwifery practices that provide primary care to all pregnant women in the Netherlands. Additionally, a collaboration took place with the Dutch influencer Midwife Mother (Dutch *Verlosmoeder*) on Instagram [31-33]. Participants filled in a survey with their first name, age, telephone number, email address, zip code, educational level, and when they planned on trying to become pregnant (currently pregnant, currently trying, or trying in ≤3, >3-12, or >12 months). Pregnant women were asked to fill in their estimated or calculated due date.

Participants were selected based on the following inclusion criteria: assigned female at birth, 18-45 years old, actively trying to become pregnant within now and 12 months or pregnant with a gestational age of <8 weeks at the start of the intervention, a low-to-intermediate educational level (prevocational or vocational education), and able and willing to download and evaluate the app.

Exclusion criteria were as follows: insufficient proficiency in the Dutch language, not in possession of a smartphone or tablet suitable for the app, and refusal to download or evaluate the app. All excluded women received a free coupon for the Dutch or English version of Smarter Pregnancy [34].

### Design

Pregnant Faster was developed by the Erasmus University Medical Center’s research group Periconception Epidemiology at the Department of Obstetrics and Gynecology, in collaboration with TJIP The Platform Engineer and the event bureau Improve. A detailed description of the cocreation and design process of Pregnant Faster and the study protocol has been published in *JMIR Protocols* [28].

### Intervention

Eligible women were sent the patient information folder, in which the intervention was explained. Inclusion was finalized after a telephone conversation in which further clarification could be provided. An email was sent with instructions on how to download and install Pregnant Faster from Apple App Store (iOS) or via a link (Android). If more than 3 days passed between inclusion and downloading, participants were approached twice by email and telephone and once by a text message to provide further support with installation.

**Figure 3:**
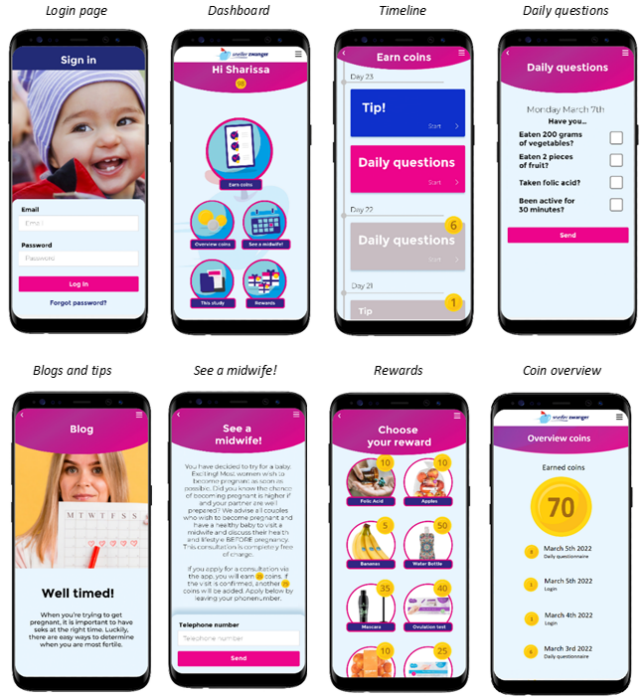
Pregnant Faster interface.

The first log-in marked the start of the 4-week intervention. During this period, participants logged in with their email address and a password, which yielded 1 coin per day per log-in. The first log-in yielded 50 coins as a reward for installation and to immediately stimulate participants to further engage with the app. After log-in, a dashboard appeared, containing 5 buttons: (1) “Earn coins,” (2) “Overview coins,” (3) “See a midwife!,” (4) “This study,” and (5) “Rewards” (Figure 3).

Button 1, “Earn coins,” led to a timeline where new blogs and tips appeared daily (Multimedia Appendix 1). Reading this information yielded 4-8 coins. In the same timeline, a daily questionnaire appeared in which participants could tick a box if they ate sufficient fruit and vegetables, exercised, and took folic acid supplements that day. Each ticked box yielded 2 coins per day. Button 2, “Overview coins,” displayed when and how coins were earned and how many coins were spent on which products. Button 3, “See a midwife!,” contained information regarding what PCC is and who it is for, stimulating participants to register through the app for a PCC consultation. Registering consisted of filling in their phone number, which immediately yielded 25 coins. An additional 75 coins were allocated after the visit was confirmed by the midwife. Button 4, “This study,” contained information about the study itself and contact details for support. Button 5, “Rewards,” contained an in-app shop where participants could order rewards, including (but not limited to) folic acid supplements, fruit, nail polish, mascara, ovulation or pregnancy tests, and newborn clothing. Rewards were sent to their home address to arrive within 5 business days. If a participant had not logged in for 7 days, they received a manually sent text message and email, encouraging them to read up on the newly offered blogs and tips, earn more coins, and order rewards.

**Figure 4:**
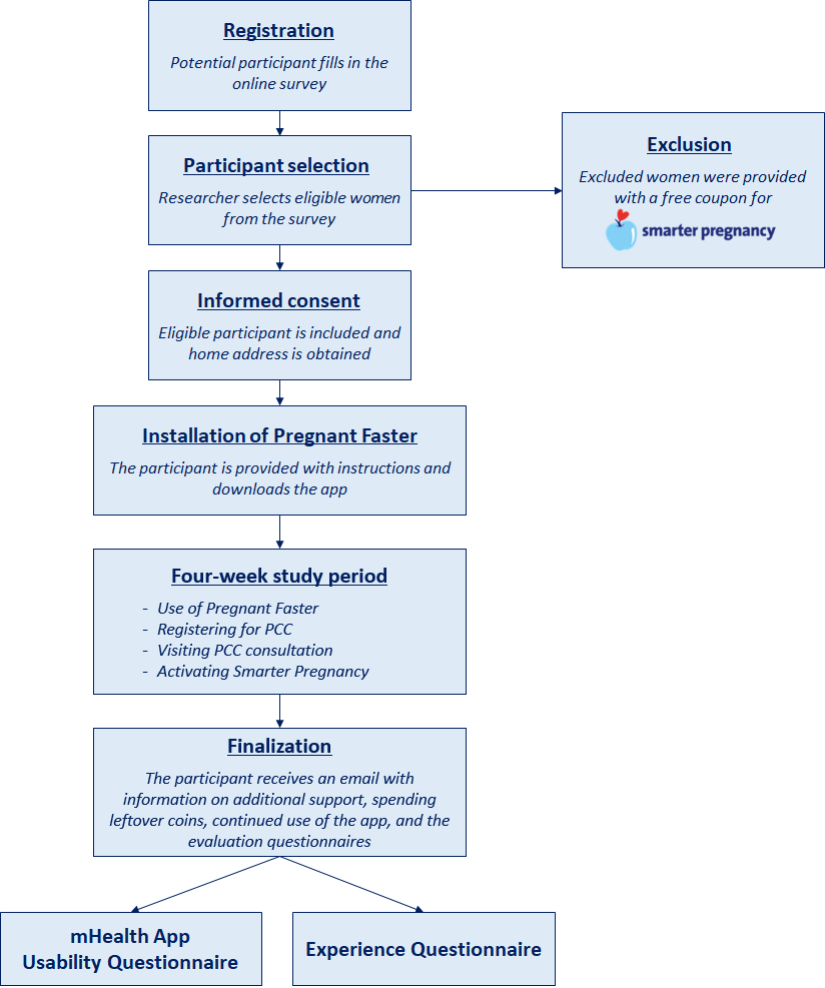
Flowchart of the study design. mHealth: mobile health; PCC: preconception care.

At the end of the intervention, participants were offered a coupon for Smarter Pregnancy via the timeline, which would yield 25 coins upon use and provide them with an additional 26 weeks of coaching. Furthermore, they received an email regarding finalization of the study and available means of support. Earned coins could be spent up to 2 weeks after the intervention ended. The blogs and tips remained accessible for as long as the app remained installed. Figure 4 provides an overview of the study flow.

### Data Collection and Outcome Measures

During the study, registration and inclusion rates were tracked and a log book was kept to note any encountered barriers and changes to the protocol.

Participants’ baseline characteristics were collected prior to inclusion through the selection survey, and home addresses were collected via email after obtaining informed consent. If selected participants did not respond to attempts to include them, they were contacted twice by email, twice by telephone, and once by a text message.

After using Pregnant Faster for 4 weeks, participants filled in a modified version of the 18-item mHealth App Usability Questionnaire (MAUQ), which uses a 7-point Likert scale (Multimedia Appendix 2) [35]. In addition, the first 10 (25.6%) participants went through a semistructured interview (Multimedia Appendix 3) in which they elaborated on their experiences with the app and, if applicable, the PCC consultation. The audiotapes of these interviews were used to compose the Experience Questionnaire (ExQ; Multimedia Appendix 4) that consisted partly of questions using a 5-point Likert scale. The ExQ was offered to the remaining participants, and the first author filled in the ExQ for the first 10 (25.6%) participants, using the audiotaped data provided during the interviews. If a question in the ExQ was not clearly answered in the interview, the participant was approached by telephone to provide an answer. The answers to the open questions in the ExQ were evaluated for notable and recurring comments.

At the end of the study period, data were collected on the number of coins earned, the types of rewards that were chosen, and the number of booked and visited PCC consultations.

### Data Analysis

To evaluate the inclusion strategy, the following percentages were calculated: (1) eligibility percentage, (2) inclusion percentages, and (3) intervention completion percentages. The eligibility percentage was determined by dividing the number of women eligible for inclusion by the total number of women who registered for the study. The inclusion percentages were calculated by dividing the number of included participants (who provided informed consent) by the number of total registrations and the number of eligible women who were approached for inclusion. Completion of the intervention entailed completing the evaluation of the app. The intervention completion percentages were obtained by dividing the number of participants who completed the intervention by the number of eligible women and the number of participants who provided informed consent.

The baseline of the study population is presented in tabular form using the median (IQR) for continuous data and n (%) for categorical data. Data obtained through the MAUQ and the ExQ are presented in bar charts. Notable answers to open questions are presented in narrative form. Data on feasibility from the researchers’ point of view are presented as bullet points.

All calculations were carried out using SPSS Statistics 25 (IBM Corporation), charts were created using Excel 2016 (Microsoft), and figures were created in PowerPoint 2016 (Microsoft).

## Results

### Recruitment, Inclusion, and the Study Population

A total of 337 women registered for the intervention, of whom 102 (30.3%) were eligible for inclusion. Informed consent was signed by 47 (46.1%) women, and 39 (83%) of the 47 women participated and completed the intervention. Table 1 displays participants’ baseline characteristics.

**Table 1:** Participants’ baseline characteristics.

Characteristics		Participants (N=39)
Age (years), median (IQR)		30 (27-35)
**Mean income neighborhood^a^, n (%)**		
	Below middle	19 (48.7)
	Above middle	11 (28.2)
	Low to high	4 (10.3)
	High	5 (12.8)
**Educational level^b^, n (%)**		
	Low	3 (7.7)
	Intermediate	36 (92.3)
**Trying to become pregnant, n (%)**		
	Currently trying	32 (82.1)
	Within 3 months	5 (12.8)
	Within 12 months	2 (5.1)
**Mobile operating system, n (%)**		
	Android	17 (43.6)
	iOS	22 (56.4)

^a^The median household income of a neighborhood is determined by the distribution of household income of all households in the country [28]. This table adheres to the original subdivision of the distribution of household income (year 2020): low, <€15,900 (<US $18,800); below middle, €15,900-21,000 (US $18,800-$24,800); middle, €21,000-26,800 (US $24,800-$31,700); above middle, €26,800-34,600 (US $31,700-$40,900); and high, >€34,600 (>US $40,900).

^b^Educational level [29]. Dutch educational levels are subdivided as follows: low (prevocational education, selective secondary education, or lower), intermediate (vocational education), and high (bachelor’s degree, master’s degree, or higher).

A 2-month gap arose between the start of recruitment and the intervention, due to the app’s CIA Triad classification [29]. A low classification was expected, but the combination of 40 intended participants and their registering their first name and email address in the app warranted a slightly higher classification for confidentiality and therefore additional security demands. Despite frequent updates to keep eligible women engaged, 55 (53.9%) of 102 women did not respond when inclusion commenced. Next, we describe these events and the inclusion process in detail.

Between September 2021 and January 2022, 212 (62.9%) women from the total 337 registrations reached in June 2022 registered for the study. Of these 212 women, only 9 (4.2%) were included. To boost the registration and inclusion rates, more flyers and posters were distributed, and the choice was made to include women who were trying to become pregnant within 12 months as opposed to within 3 months, as originally intended. Furthermore, the intervention was expanded from the municipality of Rotterdam to nationwide, delivering rewards through the postal service instead of by car. Midwives throughout the Netherlands were actively approached to ask whether they were interested in participating in the study and were offered support in setting up PCC consultations in their practices. Subsequently, the collaboration with Midwife Mother was renewed, who uploaded another post and multiple stories regarding PCC and Pregnant Faster to her Instagram. All women who were previously excluded based on not living near Rotterdam were contacted and asked to participate.

These efforts showed a limited effect. By May 2022, an additional 54 (16%) registrations and a total of 16 (34%) inclusions were obtained, which led to the decision to drop the inclusion criterion of living in a deprived neighborhood, thereby lowering the chance of including women likely to have a VHS. This choice was made to allow for further development and testing of Pregnant Faster while searching for a more effective way to recruit the intended target group for the planned larger cohort. Another social media campaign was conducted, and all women who were previously excluded based on their neighborhood’s median income were invited to participate.

Between May 2022 and July 2022, 71 (21%) more women registered for the study, adding up to the total of 337 registrations. Registration was closed after no new women registered for 2 weeks. From May onward, 31 participants were included, adding up to a total of 47 inclusions, of which 39 (83%) completed the intervention and 8 (17%) dropped out. Of these 8, 3 (37.5%) women who provided informed consent did not respond to instructions on how to install the app or attempts to reach them; 2 (25%) women were unable to install the app: in one case, an iPhone with Belgian settings preventing download from the Dutch Apple Store, and in the other case, the woman who had an Android device was scared by the warning prompted by download of an app outside of Google Play Store. Of the 3 (37.5%) remaining dropouts, 2 (25%) stopped trying to become pregnant and 1 (12.5%) found the questionnaires too burdensome. Table S1 in Multimedia Appendix 5 provides an overview of the eligibility, inclusion, and intervention completion percentages.

### PCC Consultations

A total of 17 (43.6%) of 39 participants registered for a PCC consultation, and 16 (41%) consultations were conducted by 9 midwifery practices. One consultation was performed via telehealth by the first author because the midwife chosen by the participant had no experience in providing PCC and did not wish to implement PCC in her practice. The participant who registered for PCC but did not attend a consultation was worried about her health care insurance not covering the costs. Stating to feel overwhelmed, she declined additional support as well as a free telehealth consultation.

The most often reported reason to register for a PCC consultation was to obtain more personalized information (14/17, 82.4%), followed by being curious about what a consultation entails in practice (6/17, 35.3%). The most frequent reason not to register for a consultation was simply not being interested in doing so (6/22, 27.3%). A visual overview of participants’ motivation regarding registration for PCC can be found in Figures S7 and S8 in Multimedia Appendix 6.

All participants who visited a PCC consultation agreed that registering through Pregnant Faster is easy (2/16, 12.5%, agree; 14/16, 87.5%, strongly agree) and were glad they had done so (3/16, 18.7%, agree; 13/16, 81.3%, strongly agree).

### Coins and Rewards

During the study, participants could earn a maximum amount of 468 coins. Together, they earned a total of 11.791 coins (mean 284, SD 109 per participant; median 276, IQR 221-358; range 79-443). In total, 344 rewards were ordered during the study period (mean 8, SD 6 per participant; median 7, IQR 3-12; range 0-22). One participant did not wish to order rewards because she was happy with “just the app.” She stated that she did not feel it was morally objectionable to be rewarded but just that she was not interested in receiving rewards.

The most popular reward was a €10 (US $10) book voucher, with 19 (48.7%) of 39 participants ordering the voucher at least once. The second-most popular reward was fruit, with 17 (43.6%) participants ordering fruit at least once and a total of 87 orders (87/344, 25.3%). Bananas were the most popular fruit, amounting to 32 (36.8%) of 87 fruit orders. The third-most popular reward was a set of 2 home pregnancy tests, with 16 (41%) participants ordering this reward at least once. Most participants ordered a reward more than once, displaying their personal preferences and satisfaction regarding their previous order. Table S2 in Multimedia Appendix 5 displays all rewards and their order frequency and percentage.

### Feasibility From Users’ Point of View

#### mHealth App Usability Questionnaire

**Figure 5:**
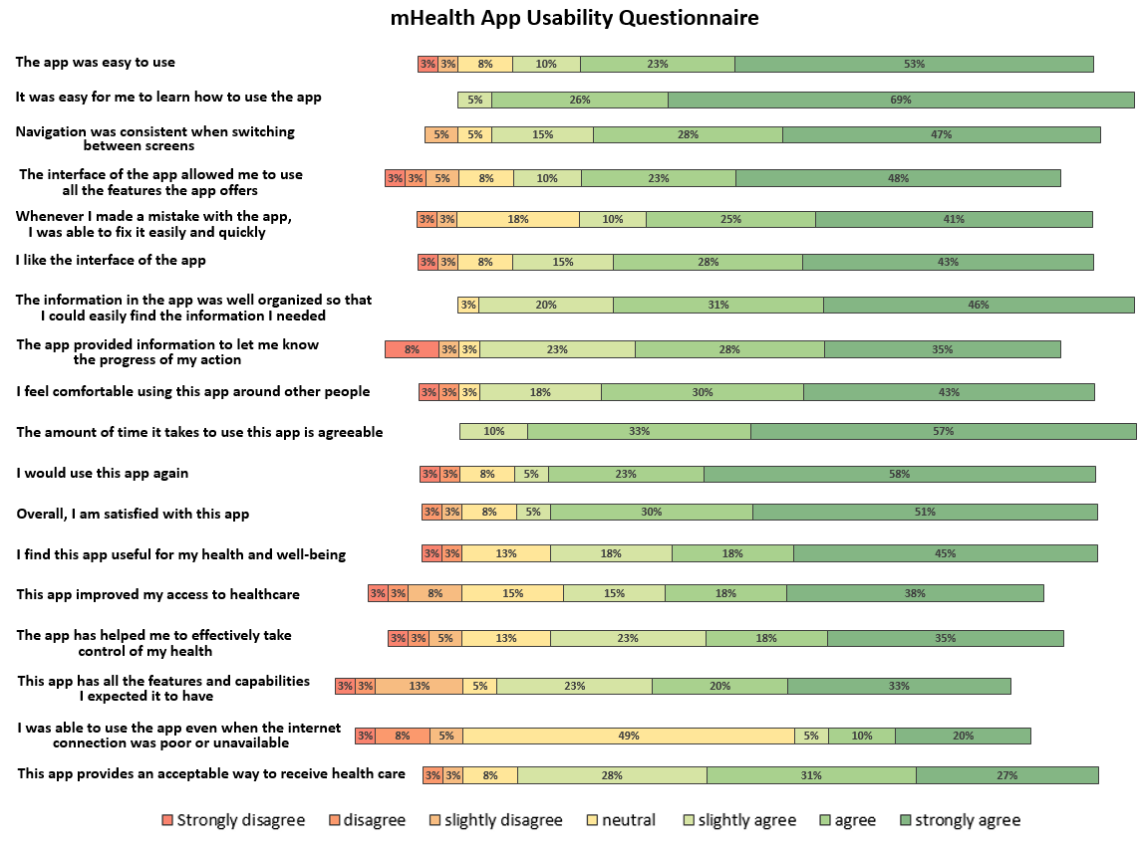
Results of the MAUQ. MAUQ: mHealth App Usability Questionnaire; mHealth: mobile health.

Figure 5 displays the results of the MAUQ. The participants deemed Pregnant Faster’s usability satisfying, with the majority of participants agreeing with 16 (88.9%) of 18 statements. With regard to the remaining 2 statements, all participants (N=39, 100%) agreed that the amount of time the app takes is agreeable, and 19 (48.7%) were neutral about being able to use the app with a poor internet connection, indicating they may not have experienced connectivity problems.

#### Experience Questionnaire

**Figure 6:**
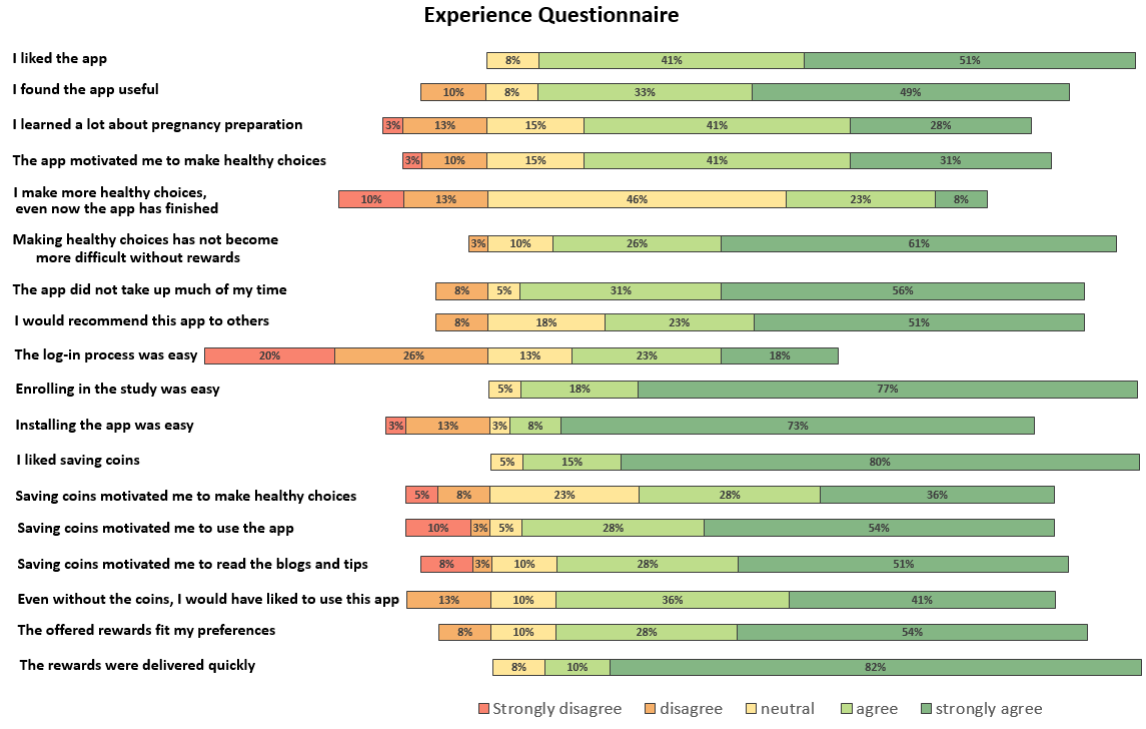
Results of the ExQ. ExQ: Experience Questionnaire.

Figure 6 displays the results of the ExQ for which the 5-point Likert scale was used. The majority of participants agreed with 16 (88.9%) of 18 statements, conveying high user satisfaction. Regarding the log-in process, 16 (41%) of 39 participants agreed and 18 (46.2%) disagreed that it is easy, with 5 (12.8%) being neutral. In addition, 12 (30.8%) participants agreed and 9 (23.1%) disagreed with the statement regarding participants making more healthy choices after finishing the intervention. The remaining 18 (46.2%) participants were neutral.

The majority of participants stated that they used the app daily (n=18, 46.2%) or every other day (n=18, 46.2%). Most participants (n=31, 79.5%) reported to have logged in less often than they would have wanted to, the foremost reason being the requirement to log in with an email and password each time (n=14, 35.9%). Overall, participants rated Pregnant Faster 7.7 out of 10, with 10 being the best rating (mean 7.7, SD 1.0; median 8, IQR 7-8; range 5-9). Multimedia Appendix 6 contains Figures S9-S11, which provide additional results for the ExQ multiple-choice questions.

In the ExQ, participants were given the option to provide additional comments. It was notable that 8 (20.5%) participants commented that they would have liked push notifications to remind them of filling in the daily questionnaire and reading new blogs and tips. One participant recommended personalized notification settings so they would best fit her wishes regarding the subject, timing, and frequency. Furthermore, 3 (7.7%) participants commented that they would like the app to focus on their partners as well, hoping to actively involve them more in preparing for pregnancy.

### Feasibility From Researchers’ Point of View

During the study, 11 issues were noted that should be considered before attempting to establish Pregnant Faster’s (cost-)effectiveness in a larger cohort study:

- A time gap between the start of recruitment and intervention led to a significant loss of eligible participants and should be prevented.
- Limiting the study population to a local area greatly impedes inclusion rates and causes disappointment in otherwise eligible participants, which may harm the intervention’s reputation.
- Using a combination of the neighborhood median income and a low-to-intermediate educational level as a proxy of low SES is not a suitable method to recruit large numbers of women with a VHS.
- Manual selection and inclusion require a significant amount of labor for which multiple researchers have to be available. The same goes for approaching individual midwifery practices for collaboration.
- Use of a classic, relatively complicated information folder and informed consent form can be overwhelming and does not lead to proper understanding of the study nor true consent.
- For participants with Android devices, installation of the app is complicated by not providing the app through the Google Play Store. Additional support is often needed.
- Fruit sent through the postal service often arrive bruised, requiring frequent checks for whether the reward is delivered in good condition, offering refunds when this is not the case. Failed deliveries result in the return of rotten fruit to the researchers. Since fruit is a popular, healthy reward, a suitable alternative should be considered, such as a voucher.
- Sending rewards daily is laborious. During this pilot, we experimented with a frequency of twice per week, clearly communicating this to participants. Afterward, no dissatisfaction regarding delivery time was noticed.
- Confirmation of PCC consultations requires the researchers to contact midwifery practices, causing a delay in coin allocation, possibly negatively impacting user satisfaction and effectiveness of the reward. Relying on participants self-reporting their visit in the app, combined with automatic coin allocation, should be considered.
- Manually keeping track of booked and confirmed consultations, in addition to manual coin allocation, requires a significant amount of time. For this reason as well, self-reporting should be considered.
- Asking participants to fill in 2 separate questionnaires causes confusion and diminishes the likeliness of completing the evaluation. It is advisable to evaluate user experiences in a succinct manner.

## Discussion

### Principal Findings

In this pilot study, we aimed to determine the feasibility of the app-based nudge Pregnant Faster, which is designed to fit the needs of women with a low SES and a high likelihood of having a VHS, who have a higher risk of adverse pregnancy outcomes. The aim of Pregnant Faster is to encourage these women by nudging them to adequately prepare for pregnancy through education by making healthy lifestyle choices and engaging in PCC, which will help improve their pregnancy outcomes.

Pregnant Faster has shown to be feasible from the users’ point of view, showing high user satisfaction with a rating of 7.7/10 and PCC uptake by 16 (41%) of 39 participants. Notably, 27 (69.2%) participants stated to have learned a lot about pregnancy preparation and 28 (71.7%) felt motivated by the app to make healthy lifestyle choices. After the intervention ended, 12 (30.8%) participants stated that they more often make healthy choices than prior to using Pregnant Faster.

With regard to the 55 (53.9%) of 102 eligible participants who did not respond when inclusion commenced after the 2-month delay, we suspect that a loss of interest and perhaps of trust in the intervention played a role. The amount of lost eligible women suggests that time is a limiting factor, impacting women’s willingness to participate in the intervention. This emphasizes the necessity of quickly responding to their willingness to participate and acceptance of offered care.

Feasibility from the researchers’ point of view was satisfactory as well but only with regard to practical conduction, as adjustments to the inclusion criteria were made to up the number of inclusions. Dropping the criterion of living in a deprived neighborhood likely impacted the chance of including women who actually have a VHS. The feasibility of Pregnant Faster from the researchers’ point of view can be improved by developing a new method of finding the target group, making the app available via both Apple App Store and Google Play Store, and automating (parts of) the inclusion process and coin allocation, which will limit the number of administrative tasks.

### Strengths and Limitations

To the best of our knowledge, Pregnant Faster is the first mHealth intervention that aims to encourage adequate pregnancy preparation and increase the uptake of PCC, promoting blended lifestyle care, by nudging participants with a loyalty program consisting of earning and spending coins. During study conduction and after evaluation, important knowledge was gained concerning the strengths and limitations of this intervention and how best to proceed with a larger cohort study.

Despite our earlier experiences regarding the recruitment of women who likely have a VHS, we did not manage to conduct this study adhering to the original inclusion criteria [28]. It is possible, therefore, that the user feasibility would have been different had the full study population met the intended criteria.

Pregnant Faster has been designed through iterative cocreation, actively involving the target population in its development [28]. Even though adjustments were made, we consider this pilot study to be another step in the iterative cocreation process, as the results will be used for further development of the app and nearly half (19/39, 48.7%) of the study population met the criteria of living in a deprived neighborhood.

### Further Development and Future Research

The insights gained through this study have prompted us to re-evaluate which characteristics to use as a proxy for a low SES and the associated VHS. To improve recruitment of the target group, we have hosted meetings with health care professionals specializing in health-related vulnerability and adverse pregnancy outcomes to gain more insight and develop new inclusion criteria for a larger cohort study. At this moment, we are researching (combinations of) different inclusion criteria based on self-reported vulnerability markers, such as high stress; financial insecurity; addiction to alcohol, drugs, and tobacco; and lack of social support, which are also associated with unfavorable health outcomes [36]. Through developing these new criteria, we aim to be more inclusive and provide support to all women with a certain degree of health-related vulnerability, instead of limiting support to those with a high likelihood of having a VHS based on the educational level and neighborhood deprivation.

We aim to continue promoting Pregnant Faster on social media platforms, such as Instagram and YouTube. These platforms have been known to use algorithms that successfully reach target audiences and prove to be effective tools with regard to providing people with support and education [37,38]. Using these platforms, therefore, will not only support recruitment and benefit the target population but also allow Pregnant Faster to contribute to pregnancy-related health in the general population.

The knowledge gained through this pilot study has inspired us to research different methods of information transfer to ensure the app fits the needs of the target group and improve Pregnant Faster’s accessibility for those who experience limited literacy [39]. For the planned cohort study, for example, we have created an audio version and infographic of the patient information folder and informed consent form. Furthermore, we are currently creating additional content for the app, again focusing on multiple methods of information transfer, such as podcasts, videos, and infographics.

On a technical level, the inclusion process and content management system will be adjusted to reduce manual tasks and promote feasibility. Furthermore, we plan to change the log-in procedure to a pin code or fingerprint and enhance the app with daily notifications.

Regarding focusing more on participants’ partners, we have chosen to not adhere to this suggestion at the current time, as the tips and blogs already contain information for partners and we are still in the process of establishing (cost-)effectiveness and further developing Pregnant Faster. In research concerning reproductive health, it is known that partners may sometimes take on a more passive role [40], which places the burden of preparing for pregnancy largely on the person who will carry the baby. For future development, therefore, we will consider the possibility of adding personalized settings to allow users to fill in characteristics that will adjust the app’s content accordingly, such as relationship status, gender and sexual orientation, and, if applicable, gestational age and the use of donor semen.

In the future, we wish to investigate the possibility of offering Pregnant Faster to all who wish to become pregnant, possibly with rewards if cost-effectiveness is established.

### Conclusion

With this pilot study, we have demonstrated that the app-based nudge Pregnant Faster provides a feasible way to stimulate the uptake of PCC and boost participants’ motivation to adequately prepare for pregnancy. We will use the knowledge we have gained through this pilot study to create an updated version of the app, which will be named Pregnant Faster 2. Our next step consists of determining the (cost-)effectiveness of Pregnant Faster 2, for which we will conduct a cohort study of 400 women with a VHS based on newly devised inclusion criteria.

## Appendix

Multimedia Appendix 1 Example of a Pregnant Faster blog.

Multimedia Appendix 2 The mHealth App Usability Questionnaire, adjusted for Pregnant Faster. mHealth: mobile health.

Multimedia Appendix 3 The Experience interview topic list.

Multimedia Appendix 4 The Experience Questionnaire.

Multimedia Appendix 5 Additional tables.

Multimedia Appendix 6 Additional figures.

## Abbreviations

CIA: confidentiality, integrity, and availability
ExQ: Experience Questionnaire
MAUQ: mHealth App Usability Questionnaire
mHealth: mobile health
PCC: preconception care
SES: socioeconomic status
VHS: vulnerable health status

## Data Availability

The data sets used in this study are available from the corresponding author upon reasonable request.

## References

[1] Timmermans S, Bonsel GJ, Steegers-Theunissen RPM, Mackenbach JP, Steyerberg EW, Raat H, Verbrugh HA, Tiemeier HW, Hofman A, Birnie E, Looman CWN, Jaddoe VWV, Steegers EAP (2011). Individual accumulation of heterogeneous risks explains perinatal inequalities within deprived neighbourhoods. Eur J Epidemiol.

[2] Hulshof KFAM, Brussaard JH, Kruizinga AG, Telman J, Löwik MRH (2003). Socio-economic status, dietary intake and 10 y trends: the Dutch National Food Consumption Survey. Eur J Clin Nutr.

[3] Beenackers MA, Kamphuis CB, Giskes K, Brug J, Kunst AE, Burdorf A, van Lenthe FJ (2012). Socioeconomic inequalities in occupational, leisure-time, and transport related physical activity among European adults: a systematic review. Int J Behav Nutr Phys Act.

[4] Steptoe A, Feldman PJ (2001). Neighborhood problems as sources of chronic stress: development of a measure of neighborhood problems, and associations with socioeconomic status and health. Ann Behav Med.

[5] Poeran J, Maas AFG, Birnie E, Denktas S, Steegers EAP, Bonsel GJ (2013). Social deprivation and adverse perinatal outcomes among Western and non-Western pregnant women in a Dutch urban population. Soc Sci Med.

[6] Vos AA, Posthumus AG, Bonsel GJ, Steegers EAP, Denktaş S (2014). Deprived neighborhoods and adverse perinatal outcome: a systematic review and meta-analysis. Acta Obstet Gynecol Scand.

[7] Waelput AJM, Sijpkens MK, Lagendijk J, van Minde MRC, Raat H, Ernst-Smelt HE, de Kroon MLA, Rosman AN, Been JV, Bertens LCM, Steegers EAP (2017). Geographical differences in perinatal health and child welfare in the Netherlands: rationale for the healthy pregnancy 4 all-2 program. BMC Pregnancy Childbirth.

[8] Agyemang C, Vrijkotte TGM, Droomers M, van der Wal MF, Bonsel GJ, Stronks K (2009). The effect of neighbourhood income and deprivation on pregnancy outcomes in Amsterdam, the Netherlands. J Epidemiol Community Health.

[9] Metcalfe A, Lail P, Ghali WA, Sauve RS (2011). The association between neighbourhoods and adverse birth outcomes: a systematic review and meta-analysis of multi-level studies. Paediatr Perinat Epidemiol.

[10] Steegers-Theunissen RPM, Twigt J, Pestinger V, Sinclair KD (2013). The periconceptional period, reproduction and long-term health of offspring: the importance of one-carbon metabolism. Hum Reprod Update.

[11] Godfrey KM, Lillycrop KA, Burdge GC, Gluckman PD, Hanson MA (2007). Epigenetic mechanisms and the mismatch concept of the developmental origins of health and disease. Pediatr Res.

[12] Hanson M, Godfrey KM, Lillycrop KA, Burdge GC, Gluckman PD (2011). Developmental plasticity and developmental origins of non-communicable disease: theoretical considerations and epigenetic mechanisms. Prog Biophys Mol Biol.

[13] van Zundert SKM, van Rossem L, Willemsen SP, van der Meer L, Ernst-Smelt HE, Steegers-Theunissen RPM (2022). Periconceptional maternal social, lifestyle and medical risk factors impair embryonic growth: the Rotterdam Periconceptional Cohort. Reprod Biomed Online.

[14] van Uitert EM, Exalto N, Burton GJ, Willemsen SP, Koning AHJ, Eilers PHC, Laven JSE, Steegers EAP, Steegers-Theunissen RPM (2013). Human embryonic growth trajectories and associations with fetal growth and birthweight. Hum Reprod.

[15] de Mendonça ELSS, de Lima Macêna M, Bueno N, de Oliveira ACM, Mello C (2020). Premature birth, low birth weight, small for gestational age and chronic non-communicable diseases in adult life: a systematic review with meta-analysis. Early Hum Dev.

[16] Lastrucci V, Lorini C, Caini S, Bonaccorsi G, Florence Health Literacy Research Group (2019). Health literacy as a mediator of the relationship between socioeconomic status and health: a cross-sectional study in a population-based sample in Florence. PLoS One.

[17] M'hamdi HI, Sijpkens MK, de Beaufort I, Rosman AN, Steegers EA (2018). Perceptions of pregnancy preparation in women with a low to intermediate educational attainment: a qualitative study. Midwifery.

[18] Wildschut HIJ, van Vliet-Lachotzki EH, Boon BM, Lie Fong S, Landkroon AP, Steegers EAP (2006). [Preconception care: an essential part of the care for mother and child]. Ned Tijdschr Geneeskd.

[19] de Weerd S, Steegers EAP, Heinen MM, van den Eertwegh S, Vehof RMEJ, Steegers-Theunissen RPM (2003). Preconception nutritional intake and lifestyle factors: first results of an explorative study. Eur J Obstet Gynecol Reprod Biol.

[20] Van Dijk MR, Huijgen NA, Willemsen SP, Laven JS, Steegers EA, Steegers-Theunissen RP (2016). Impact of an mHealth platform for pregnancy on nutrition and lifestyle of the reproductive population: a survey. JMIR Mhealth Uhealth.

[21] Poels M, Koster MPH, Boeije HR, Franx A, van Stel HF (2016). Why do women not use preconception care? A systematic review on barriers and facilitators. Obstet Gynecol Surv.

[22] van Dijk MR, Koster MPH, Oostingh EC, Willemsen SP, Steegers EAP, Steegers-Theunissen RPM (2020). A mobile app lifestyle intervention to improve healthy nutrition in women before and during early pregnancy: single-center randomized controlled trial. J Med Internet Res.

[23] Gootjes DV, van Dijk MR, Koster MP, Willemsen SP, Steegers EA, Steegers-Theunissen RP (2019). Neighborhood deprivation and the effectiveness of mobile health coaching to improve periconceptional nutrition and lifestyle in women: survey in a large urban municipality in the Netherlands. JMIR Mhealth Uhealth.

[24] Smith SM, van der Kleij RMJJ, Bais B, Schermer MHN, Ismaili M'hamdi H, Steegers-Theunissen RPM (2022). Preferences of women with a vulnerable health status towards nudging for adequate pregnancy preparation as investment in health of future generations: a qualitative study. BMC Pregnancy Childbirth.

[25] Thaler R, Sunstein C (2008). Nudge : improving decisions about health, wealth, and happiness. New Haven, Conn.

[26] Murayama H, Takagi Y, Tsuda H, Kato Y (2023). Applying nudge to public health policy: practical examples and tips for designing nudge interventions. Int J Environ Res Public Health.

[27] van der Windt M, van der Kleij RM, Snoek KM, Willemsen SP, Dykgraaf RHM, Laven JSE, Schoenmakers S, Steegers-Theunissen RPM (2020). Impact of a blended periconception lifestyle care approach on lifestyle behaviors: before-and-after study. J Med Internet Res.

[28] Smith SM, Bais B, Ismaili M'hamdi H, Schermer MHN, Steegers-Theunissen RPM (2023). Stimulating the uptake of preconception care by women with a vulnerable health status through mHealth app-based nudging (Pregnant Faster): cocreation design and protocol for a cohort study. JMIR Res Protoc.

[29] Singh K, Rishiwal V, Kumar P (2018). Classification of data to enhance data security in cloud computing.

[30] Would you like to become pregnant?. Sneller Zwanger.

[31] VerlosMoeder online platform. VerlosMoeder.

[32] VerlosMoeder. YouTube.

[33] Djanifa da Conceicao. Instagram.

[34] (2019). Smarter Pregnancy: for couples who are or wish to get pregnant. Erasmus University Medical Center.

[35] Zhou L, Bao J, Setiawan IMA, Saptono A, Parmanto B (2019). The mHealth App Usability Questionnaire (MAUQ): development and validation study. JMIR Mhealth Uhealth.

[36] Aday LA (1994). Health status of vulnerable populations. Annu Rev Public Health.

[37] Carpenter J, Morrison S, Craft M, Lee M (2020). How and why are educators using Instagram?. Teach Teach Educ.

[38] Shoufan A, Mohamed F (2022). YouTube and education: a scoping review. IEEE Access.

[39] Seligman HK, Wallace AS, DeWalt DA, Schillinger D, Arnold CL, Shilliday BB, Delgadillo A, Bengal N, Davis TC (2007). Facilitating behavior change with low-literacy patient education materials. Am J Health Behav.

[40] Pachauri S (2001). Male involvement in reproductive health care. J Indian Med Assoc.

