# Supplementary material for: Stimulating Preconception Care Uptake by Women With a Vulnerable Health Status Through a Mobile Health App (Pregnant Faster): Pilot Feasibility Study
Source: JMIR Hum Factors. 2024 Apr 22;11:e53614. doi: 10.2196/53614 (PMC11074886; doi:10.2196/53614)
Supplement: Multimedia Appendix 1 [file humanfactors_v11i1e53614_app1.docx]

# Multimedia Appendix 1

### Example of a Pregnant Faster blog

| **Preconception care**  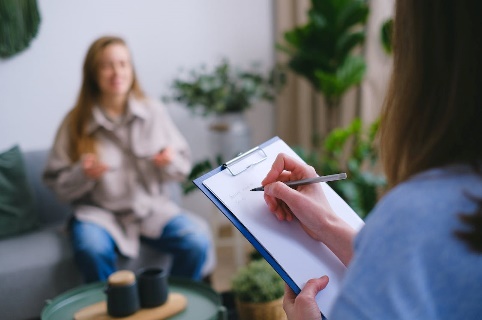  Have you ever heard of pregnancy care prior to pregnancy? Now you have! Preconception care is meant for women and couples who plan to become pregnant within the next 6 months and aims to help you increase your chance to become pregnant and have a healthy baby. And the best part; Preconception care is fully covered by your insurance.  During a preconception care consultation, a midwife or gynaecologists discusses matters that affect your health and that of your (future) baby. For instance, how healthy your lifestyle choices are, your profession and of course whether you have already started taking folic acid.  Additionally, a swift physical check-up is performed, checking things such as your blood pressure and glucose level. During this consultation you can ask all sorts of questions related to having a baby, to which you receive highly personalized answers. Do you have an IUD? If so, IUDs can be removed during this consultation as well.  Did you know that eight out of ten women who want to have children could do at least one thing to get pregnant faster and healthier? And that the average woman has 5 points that she can pay close attention to? Yep, plenty of reason to sign up! If you register for a consultation via the 'Visit a midwife' button in this app, you will earn a total of 100 coins! More than enough to order folic acid and fruit through the ‘Rewards’ button and get you started nicely.  And don’t worry! Preconception care is not a check-up to make sure you are fit to become a parent. We’re here to help you get the most out of this journey. No one has to be perfect and every small change counts!  If you have any questions about preconception care, send us an email ([snellerzwanger@erasmusmc.nl](mailto:snellerzwanger@erasmusmc.nl)) or a text message (+316 xxx xxx xx). We’re happy to help you on your way! |
| --- |
