# Supplementary material for: Stimulating Preconception Care Uptake by Women With a Vulnerable Health Status Through a Mobile Health App (Pregnant Faster): Pilot Feasibility Study
Source: JMIR Hum Factors. 2024 Apr 22;11:e53614. doi: 10.2196/53614 (PMC11074886; doi:10.2196/53614)
Supplement: Multimedia Appendix 2 [file humanfactors_v11i1e53614_app2.docx]

# Multimedia Appendix 2

### The mHealth App Usability Questionnaire – adjusted for *Pregnant Faster*

Answer options 🡪 7-point Likert scale

I strongly agree | I agree | I slightly agree | Neutral | I slightly disagree | I disagree | I strongly disagree

Questions

The app was easy to use

It was easy for me to learn how to use the app

Navigation was consistent when switching between screens

The interface of the app allowed me to use all the features the app offers (e.g. enter information, respond to reminders, view information)

Whenever I made a mistake with the app, I was able to fix it easily and quickly

I like the interface of the app

The information in the app was well organized so that I could easily find the information I needed

The app provided information to let me know the progress of my action

I feel comfortable using this app around other people

The amount of time it takes to use this app agrees with me

I would use this app again

Overall, I am satisfied with this app

I find this app useful for my health and well-being

This app improved my access to healthcare

The app has helped me to effectively take control of my health

This app has all the features and capabilities I expected it to have

I was able to use the app even when the internet connection was poor or unavailable

This app provides an acceptable way to receive health care, such as access to educational materials, tracking my own activities, and conducting self-assessments
