# Supplementary material for: Stimulating Preconception Care Uptake by Women With a Vulnerable Health Status Through a Mobile Health App (Pregnant Faster): Pilot Feasibility Study
Source: JMIR Hum Factors. 2024 Apr 22;11:e53614. doi: 10.2196/53614 (PMC11074886; doi:10.2196/53614)
Supplement: Multimedia Appendix 4 [file humanfactors_v11i1e53614_app4.docx]

# Multimedia Appendix 4

### The Experience Questionnaire

Question A

Options (one answer): strongly disagree, disagree, neutral, agree, strongly agree

1. I liked the app
2. I found the app useful
3. I learned a lot about pregnancy preparation
4. The app motivated me to make healthy choices
5. I make more healthy choices, even now the app has finished
6. Making healthy choices has not become more difficult now that I do not

receive rewards anymore

1. The app did not take up much of my time
2. I would recommend this app to others
3. The log-in process was easy
4. Enrolling in the study was easy
5. Installing the app was easy
6. I liked saving coins
7. Saving coins motivated me to make healthy choices
8. Saving coins motivated me to use the app
9. Saving coins motivated me to read the blogs and tips
10. Even without the coins, I would have liked to use this app
11. The offered rewards fit my preferences
12. The rewards were delivered quickly
13. Registering for a preconception care consultation was easy through the app
14. I am glad I visited a preconception care consultation

Question B

I logged in less often than I wanted to, mainly due to (one answer):

1. Having to log-in with email and password
2. Having too little time
3. Forgetting I had the app
4. Not applicable. I did not log in less than I wanted to

Question C

I have used the app (one answer)

1. Daily
2. Every other day
3. Twice a week
4. Once a week
5. Less than once a week

Question D

My foremost reason not to register for a preconception care consultation was (one answer):

1. Just not being interested in doing so
2. Not noticing the option for registration in the app
3. Not understanding why it would be useful for me
4. Feeling it was too demanding
5. Feeling it did not apply to me because I am undergoing fertility treatment
6. Feeling it made trying for a baby ‘too real’
7. I have recently visited a preconception care consultation

Question E

My reasons to register for a preconception care consultation were (multiple answers):

1. I wanted more personalized information
2. I wanted to earn coins
3. I was curious to see what it entailed in practice
4. I thought it would be fun to do

Question F

Out of 10, I rate this app: …

1 (most unsatisfied) – 10 (perfect)

Question G

I used the app on the operating system (one answer):

1. Android
2. iOS (Apple iPhone)
