# Supplementary material for: Stimulating Preconception Care Uptake by Women With a Vulnerable Health Status Through a Mobile Health App (Pregnant Faster): Pilot Feasibility Study
Source: JMIR Hum Factors. 2024 Apr 22;11:e53614. doi: 10.2196/53614 (PMC11074886; doi:10.2196/53614)
Supplement: Multimedia Appendix 5 [file humanfactors_v11i1e53614_app5.docx]

# Multimedia Appendix 5

## Additional tables

**Table S1. Recruitment and inclusion rates**

|  | **ɳ** | **%** |  |
| --- | --- | --- | --- |
| **Number of registrations** | 337 |  |  |
| **Eligible women** | 102 | 30.3% | of 337 total registrations |
| **Included participants** | 47 | 13.9% | of 337 total registrations |
|  |  | 46.1% | of 102 eligible women |
| **Number of completed interventions** | 39 | 38.2% | of 102 eligible women |
|  |  | 83% | of 47 included participants |

**Table S2. Overview of rewards**

|  | **ɳ of participants** | **% of participants (ɳ=39)** | **ɳ of total orders** | **% of total rewards (ɳ=344)** |
| --- | --- | --- | --- | --- |
| **Book voucher (€10.00 - $10.00)** | 19 | 49% | 47 | 13.7% |
| **Fruit** | 17 | 44% | 87 | 25.3% |
| **Bananas (5)** | 15 | 39% | 32 | 9.3% |
| **Grapes (1 box)** | 13 | 33% | 27 | 7.8% |
| **Tangerines (8)** | 10 | 26% | 13 | 3.8% |
| **Apples (8)** | 9 | 23% | 15 | 4.4% |
| **Home pregnancy tests (2)** | 16 | 41% | 31 | 9.0% |
| **Folic acid supplements** | 16 | 41% | 24 | 7.0% |
| **New born clothing set** | 14 | 36% | 21 | 6.1% |
| **Facial mask** | 12 | 31% | 36 | 10.5% |
| **Mascara** | 11 | 28% | 15 | 4.4% |
| **Water bottle** | 9 | 23% | 12 | 3.5% |
| **New born romper** | 8 | 21% | 10 | 2.9% |
| **Ovulation detection tests (5)** | 7 | 18% | 10 | 2.9% |
| **Vegan meal (1 person)** | 7 | 18% | 10 | 2.9% |
| **Nail polish** | 6 | 15% | 8 | 2.3% |
| **Resistance bands (set of 5)** | 6 | 15% | 6 | 1.7% |
| **BIBS pacifier** | 5 | 13% | 7 | 2.0% |
| **LUSH wash card** | 4 | 10% | 7 | 2.0% |
| **New born hat** | 3 | 8% | 3 | 0.9% |
