# Supplementary material for: Stimulating Preconception Care Uptake by Women With a Vulnerable Health Status Through a Mobile Health App (Pregnant Faster): Pilot Feasibility Study
Source: JMIR Hum Factors. 2024 Apr 22;11:e53614. doi: 10.2196/53614 (PMC11074886; doi:10.2196/53614)
Supplement: Multimedia Appendix 6 [file humanfactors_v11i1e53614_app6.docx]

# Multimedia Appendix 6

## Additional figures

**Figure S7. Participants’ reasons for registering for a PCC consultation**

**Figure S8. Participants’ reasons for not registering for a PCC consultation**

**
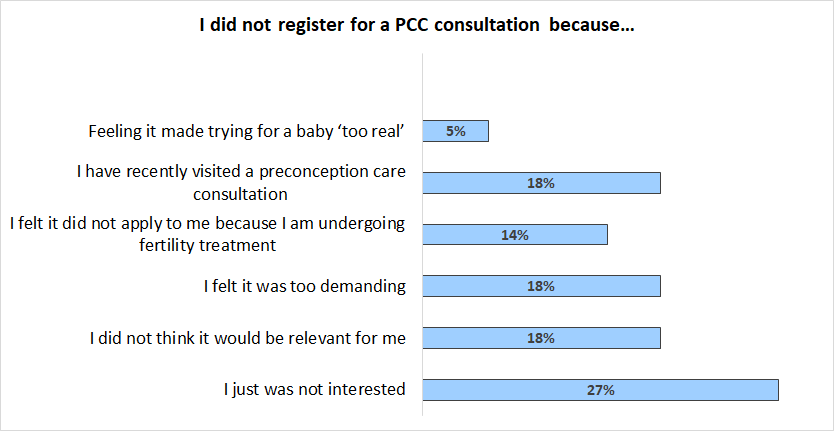
**

**Figure S9. Participant’s frequency of using Pregnant Faster**

**Figure S10. Participants’ reasons for logging-in less often than they wanted**

**Figure S11. Participants’ rating of Pregnant Faster**
